# Hypo-Substitute for Opiates

**Published:** 1899-03

**Authors:** 


### HYPO-SUBSTITUTE FOR OPIATES.

Dr. Obe F. Watlington, of Memphis Tenn., writes in the *Medical Brief*, "I have in my possession a hypodermic alkaloidal solution which is a specific in drug addictions (opium habituation, alcoholism, etc). On receipt of a two-cent stamp I will take pleasure in furnishing any physician the formula by the use of which a number of the fraternity have been enabled to cure themselves of opiumism, alcoholism, and insomnia. I used morphia hypodermically for ten years. Obtained a perfect cure by this prescription."

